# Short-term overnutrition induces white adipose tissue insulin resistance through *sn*-1,2-diacylglycerol/PKC**ε**/insulin receptor Thr^1160^ phosphorylation

**DOI:** 10.1172/jci.insight.139946

**Published:** 2021-02-22

**Authors:** Kun Lyu, Dongyan Zhang, Joongyu Song, Xiruo Li, Rachel J. Perry, Varman T. Samuel, Gerald I. Shulman

**Affiliations:** 1 Department of Internal Medicine, Yale School of Medicine, New Haven, Connecticut, USA.; 2 Department of Cellular & Molecular Physiology, Yale School of Medicine, New Haven, Connecticut, USA.; 3 VA Connecticut Healthcare System, West Haven, Connecticut, USA.

**Keywords:** Endocrinology, Metabolism, Adipose tissue, Glucose metabolism, Insulin signaling

## Abstract

White adipose tissue (WAT) insulin action has critical anabolic function and is dysregulated in overnutrition. However, the mechanism of short-term high-fat diet–induced (HFD-induced) WAT insulin resistance (IR) is poorly understood. Based on recent evidences, we hypothesize that a short-term HFD causes WAT IR through plasma membrane (PM) *sn*-1,2-diacylglycerol (*sn*-1,2-DAG) accumulation, which promotes protein kinase C-ε (PKCε) activation to impair insulin signaling by phosphorylating insulin receptor (Insr) Thr^1160^. To test this hypothesis, we assessed WAT insulin action in 7-day HFD–fed versus regular chow diet–fed rats during a hyperinsulinemic-euglycemic clamp. HFD feeding caused WAT IR, reflected by impaired insulin-mediated WAT glucose uptake and lipolysis suppression. These changes were specifically associated with PM *sn*-1,2-DAG accumulation, higher PKCε activation, and impaired insulin-stimulated Insr Tyr^1162^ phosphorylation. In order to examine the role of Insr Thr^1160^ phosphorylation in mediating lipid-induced WAT IR, we examined these same parameters in Insr^T1150A^ mice (mouse homolog for human Thr^1160^) and found that HFD feeding induced WAT IR in WT control mice but not in Insr^T1150A^ mice. Taken together, these data demonstrate the importance of the PM *sn*-1,2-DAG/PKCε/Insr Thr^1160^ phosphorylation pathway in mediating lipid-induced WAT IR and represent a potential therapeutic target to improve WAT insulin sensitivity.

## Graphical abstract

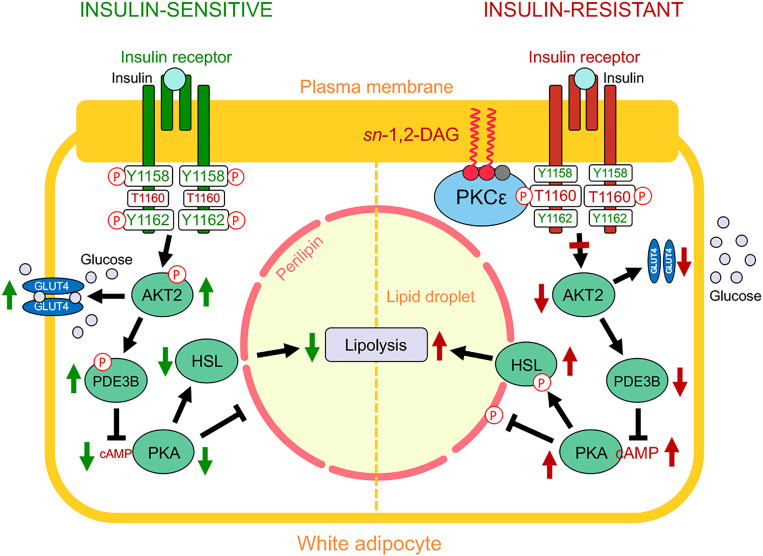

## Introduction

Obesity-related metabolic diseases such as type 2 diabetes and metabolic-associated fatty liver disease (MAFLD) are often accompanied by white adipose tissue (WAT) dysfunction (1, 2). One aspect of WAT dysfunction is WAT insulin resistance, which is partially characterized by insulin’s reduced ability to suppress lipolysis, resulting in higher rates of fatty acid delivery to liver and skeletal muscle (1, 3). Ectopic lipid accumulation in insulin-responsive tissues such as liver and skeletal muscle leads to insulin resistance via the accumulation of diacylglycerol (DAG) in the plasma membrane (PM) and subsequent translocation and activation of novel PKCs (nPKCs) (1, 4, 5). Accumulation of *sn*-1,2-DAGs in the PM activates protein kinase C-ε (PKCε), which then phosphorylates insulin receptor (Insr) at Thr^1160^ to impair Insr kinase (IRK) autophosphorylation/activation and subsequent activation of downstream signaling events (1, 5–7). This simple model can explain the development of lipid-induced liver and muscle insulin resistance in obese rodents and humans (1, 4–10), as well as the mechanism by which weight loss (11), adiponectin (12), and liver-targeted mitochondrial uncouplers reverse insulin resistance in HFD-fed obese (13, 14) and lipodystrophic (15) insulin–resistant rodents.

The pathogenesis of WAT insulin resistance remains unclear. Multiple factors have already been implicated, including low-grade inflammation, altered adipokine secretion, and hypoxia (16–18). However, further studies have suggested that WAT insulin resistance can develop early following overfeeding as a primary event. Additionally, overfed human subjects can develop peripheral insulin resistance prior to WAT immune cell infiltration (19, 20).

Similar to its critical role in lipid-induced hepatic insulin resistance, PKCε also appears to be important in the pathogenesis of WAT insulin resistance. Silencing of both hepatic and WAT PKCε with an antisense oligonucleotide improved WAT insulin action, reflected by increased insulin-stimulated WAT glucose uptake in high-fat diet–fed (HFD-fed) rats (5). Consistent with these results, Brandon and colleagues recently demonstrated improvement of glucose tolerance in HFD-fed WAT-specific PKCε-KO mice (21). However, they did not document any alterations in the WAT insulin signaling pathway or in insulin’s suppression of WAT lipolysis in these mice. Furthermore, there was no conclusion on whether the DAG/PKCε/Insr Thr^1160^ pathway was involved in WAT insulin action regulation or whether another distinct molecular pathway is regulated by PKCε activation to impact glucose tolerance. In this study, we examine the hypothesis that the DAG/PKCε/Insr Thr^1160^ pathway may be an early contributor to short-term HFD–induced WAT insulin resistance and hinder the ability of insulin to suppress WAT lipolysis and promote WAT glucose uptake, which is necessary for fatty acid esterification into triglyceride.

In order to test this hypothesis, we first explored this pathway in male Sprague Dawley rats fed with HFD versus regular chow (RC) diet for 7 days, a time frame in which fat feeding may induce WAT insulin resistance without causing WAT inflammation in rats (16). We measured the content of DAG stereoisomers in 5 different subcellular compartments (PM, ER, mitochondria [Mito], cytosol, and lipid droplet [LD]), and assayed the translocation of different PKCs, and the activation of key steps of the insulin signaling pathway. We further quantified the impact of WAT insulin resistance with a hyperinsulinemic-euglycemic clamp (HEC) study, using a combination of stable and radiolabeled isotope tracers to measure the alterations in whole-body and WAT fatty acid flux and glucose metabolism. Furthermore, to determine whether phosphorylation of Insr Thr^1160^ is necessary for lipid-induced WAT insulin resistance, we performed the same HEC with stable and radiolabeled isotope tracers in WT versus Insr^T1150A^ knock-in mice on HFD to assess their WAT insulin sensitivity using these same methods.

## Results

### Seven-day HFD causes WAT insulin resistance.

**Figure 1:**
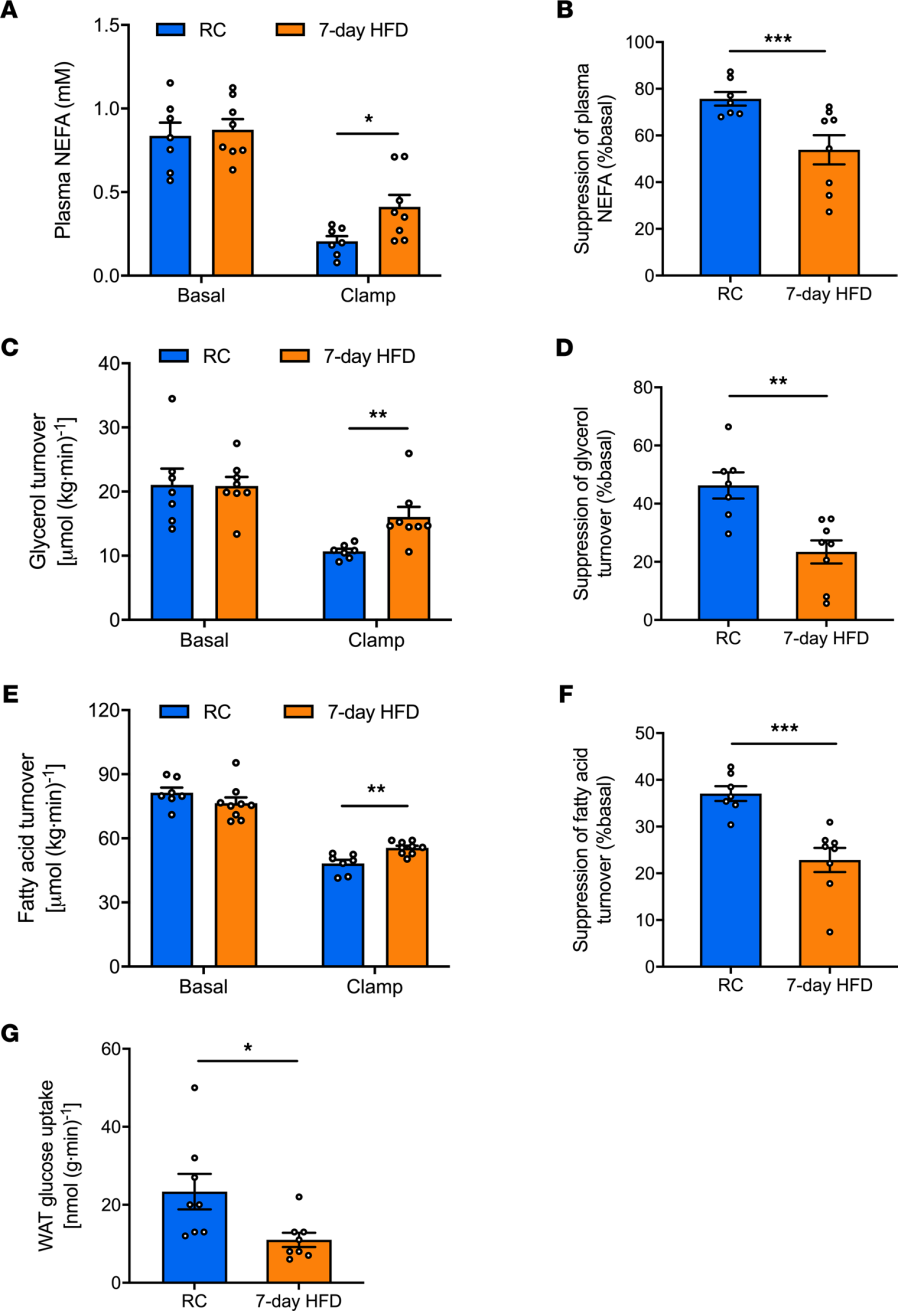
Seven-day HFD causes WAT insulin resistance reflected by reductions in WAT glucose uptake and insulin’s suppression of WAT lipolysis. (**A**) Plasma NEFA under basal (overnight fasting) and hyperinsulinemic-euglycemic clamp conditions. (**B**) Insulin’s suppression of plasma NEFA during the clamp. (**C** and **D**) Whole-body glycerol turnover and its suppression by insulin during the hyperinsulinemic-euglycemic clamp. (**E** and **F**) Whole-body fatty acid turnover and its suppression by insulin during the hyperinsulinemic-euglycemic clamp. (**G**) Insulin-stimulated WAT glucose uptake. In all panels, data are the mean ± SEM of *n* = 7–10 per group, with comparisons by 2-tailed unpaired Student’s *t* test. *P < 0.05, **P < 0.01, ***P < 0.001.

We performed a HEC combined with [^2^H_7_]glucose, [^2^H_5_]glycerol, and [^13^C_16_]palmitate infusions in male Sprague Dawley rats fed either RC or a 7-day HFD (Supplemental Figure 1, A–D; supplemental material available online with this article; https://doi.org/10.1172/jci.insight.139946DS1). There was no significant difference in body weight, fasting plasma glucose, or insulin concentrations between the RC and HFD group (Supplemental Table 1). We quantified fatty acid and glycerol turnover in the basal (fasted) state and 30 minutes after a primed (25 mU/[kg-min] × 5 minutes), continuous (2.5 mU/[kg-min]) insulin infusion. This short HEC clamp was sufficient to establish a steady state in fatty acid and glycerol turnover (Supplemental Figure 1, E and F), and it allowed us to measure changes in insulin action and signaling linked to the suppression of WAT lipolysis. There were no significant differences in fasting plasma nonesterified fatty acids (NEFA) concentrations or rates of whole-body lipolysis between the groups (Figure 1, A, C, and E). However, after 7-day HFD, the ability of insulin to suppress WAT lipolysis was impaired, and we observed less suppression of NEFA concentration (Figure 1, A and B). Consistent with these results, we found that insulin suppression of whole-body glycerol turnover and fatty acid turnover during the HEC clamp were also impaired with 7-day HFD feeding (Figure 1, C–F, and Supplemental Figure 1, C and D). Furthermore, we performed a 140-minute HEC to quantify insulin-stimulated glucose uptake in WAT. Rats fed a 7-day HFD exhibit an approximately 50% reduction in insulin-stimulated WAT glucose uptake (Figure 1G).

In summary, 7 days of HFD feeding caused WAT insulin resistance, reflected by reductions in insulin-mediated WAT glucose uptake and suppression of WAT lipolysis.

### Seven-day HFD impairs insulin-stimulated insulin signaling cascade activation in WAT.

**Figure 2:**
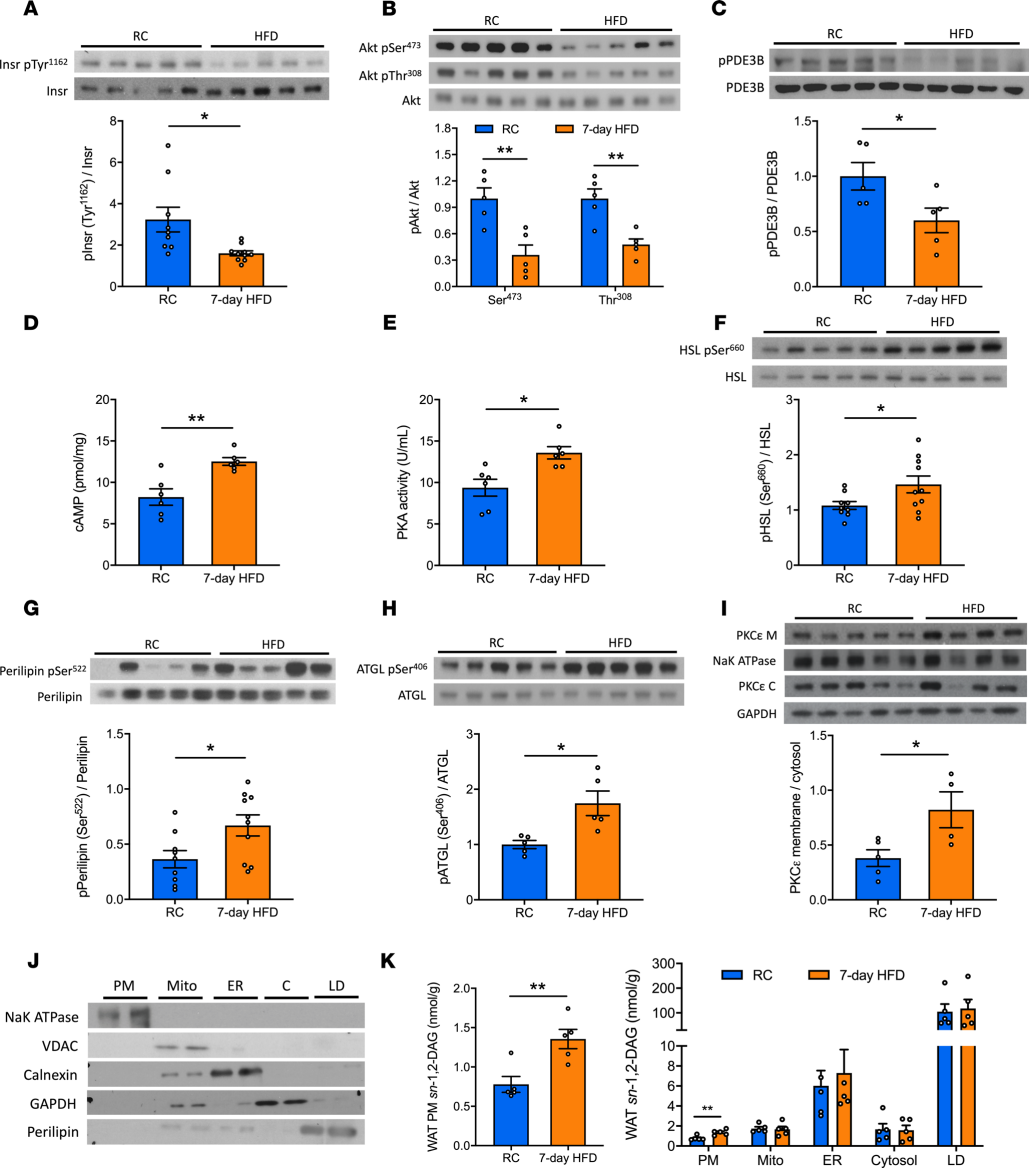
Seven-day HFD feeding impairs insulin-stimulated insulin signaling cascade in WAT associated with increases in plasma membrane *sn*-1,2-DAGs and PKCε translocation. (**A**–**C**) Insulin-stimulated phosphorylation of Insr, Akt, and PDE3B in WAT. (**D** and **E**) WAT cAMP and PKA activity during the hyperinsulinemic-euglycemic clamp. (**F**–**H**) Insulin-stimulated phosphorylation of HSL, perilipin, and ATGL. (**I**) WAT PKCε membrane/cytosol ratio. (**J**) Separation of 5 subcellular compartments in WAT: plasma membrane (PM), mitochondria (Mito), ER, cytosol (C), and lipid droplet (LD). (**K**) WAT *sn*-1,2-DAGs in 5 compartments. In **A**–**H**, rats (after overnight fasting) were under hyperinsulinemic-euglycemic clamp conditions. Data are the mean ± SEM of *n* = 5–10 per group. In **I**–**K**, rats were under 6-hour fasting basal condition; data are the mean ± SEM of *n* = 4–5 per group. In all panels, groups are compared by 2-tailed unpaired Student’s *t* test. *P < 0.05, **P < 0.01.

We next explored potential mechanisms by which HFD impairs insulin’s suppression of WAT lipolysis by examining the components of the insulin signaling cascade that regulate the key lipolytic enzymes in epididymal WAT. Insulin-stimulated phosphorylation of both Insr and Akt were decreased in HFD-fed rats compared with the RC group (Figure 2, A and B), suggesting that the defect in insulin action could be attributed to impaired IRK activation. The canonical pathway by which insulin suppresses WAT lipolysis is mainly through activation of phosphodiesterase 3B (PDE3B), which then degrades cAMP to halt the activation of protein kinase A–mediated (PKA-mediated) phosphorylation of lipolytic enzymes, leading to decreased activity of hormone-sensitive lipase (HSL) and reduced LD protection from perilipin. Thus, we measured insulin-stimulated PDE3B phosphorylation, cAMP content, and PKA activity. Rats subjected to 7-day HFD exhibited decreased insulin-stimulated phosphorylation of PDE3B, and this was associated with higher cAMP concentrations and PKA activity in WAT (Figure 2, C–E). These changes were associated with increased phosphorylation of key lipolytic proteins — adipose triglyceride lipase (ATGL) at Ser^406^, hormone-sensitive lipase (HSL) at Ser^660^, and perilipin at Ser^522^ (Figure 2, F–H). Basal levels of these key WAT insulin signaling proteins were unchanged (Supplemental Figure 2E).

In summary, 7 days of HFD feeding impaired insulin signaling in WAT at the level of Insr autophosphorylation, limiting the ability of insulin to decrease activity of the downstream effectors of WAT lipolysis.

### Seven-day HFD increases WAT PM sn-1,2-DAG content and PKCε translocation.

Both HFD-induced WAT insulin resistance and hepatic insulin resistance have an insulin signaling defect at the level of IRK activation. In the liver, impaired IRK activation is attributed to activation of PKCε, and we hypothesized that a short-term HFD may also lead to activation of PKCε in WAT. Since there is little data on the role of PKCs in regulating WAT physiology, we first assayed the activation of certain major PKC isoforms. Specifically, we measured translocation (from cytosol to membrane, an index of activation) of both conventional and nPKC isoforms in WAT in RC and 7-day HFD–fed rats. The translocation and activation of PKCε, as reflected by the membrane/cytosol ratio of PKCε, increased by about 2-fold in HFD-fed rats versus the RC-fed group (Figure 2I). In contrast, the membrane translocation of other PKC isoforms, including α, β, θ, and δ, were unaltered by the 7-day HFD feeding (Supplemental Figure 3, A–D). PKCs are activated by DAGs — specifically, *sn*-1,2-DAGs (22). DAGs are present in multiple cellular compartments, such as the ER, Mito, PM, LD, and cytosol. Thus, we developed an assay to separate 5 subcellular compartments in WAT (Figure 2J). Next, we measured the concentration of DAG stereoisomers in each fraction. As expected, approximately 90% of total DAG was located in the LD fraction (Supplemental Figure 2A). There were no differences in total DAG concentrations between the groups (Supplemental Figure 2B). However, we observed an approximately 2-fold increase in *sn*-1,2-DAGs in the PM compartment, with no difference observed in the other 2 DAG stereoisomers (Figure 2K; Supplemental Figure 2, C and D; and Supplemental Table 3). These changes occurred without evidence of adipose inflammation. Expression of genes associated with WAT inflammation and hypoxia were unchanged (Supplemental Figure 3E).

Taken together, these data suggest that activation of the PKCε pathway, by a short-term HFD, is triggered by PM-associated *sn*-1,2-DAG accumulation and that the *sn*-1,2-DAG/PKCε pathway may be the primary driver of impaired WAT insulin signaling in short-term overnutrition.

### Insr^T1150A^ mice are protected from a 7-day HFD–induced WAT insulin resistance.

We had previously identified Insr Thr^1160^ as a specific residue that is phosphorylated by PKCε in the liver (6). Phosphorylation of this residue decreases the tyrosine kinase activity of Insr and downstream signaling events. Mutation of this residue from a threonine to an alanine (i.e., Insr^T1150A^) shields Insr from this pathogenic phosphorylation and preserves hepatic insulin signaling and hepatic insulin sensitivity in HFD-fed mice. In our previous study, we did not observe any protective effects on WAT insulin action in HFD-fed Insr^T1150A^ mice (6). However, these assessments of WAT metabolism were performed during the final stages of a 140-minute HEC with an insulin infusion rate at 2.5 mU/(kg•min). Suppression of WAT lipolysis occurs rapidly after the onset of hyperinsulinemia (16), and the degree of WAT insulin resistance after just several days of HFD feeding is subtle and can be surmounted with high plasma insulin concentrations. Thus, any differences in WAT lipolysis may have been obscured in our previous studies involving the Insr^T1150A^ mice.

**Figure 3:**
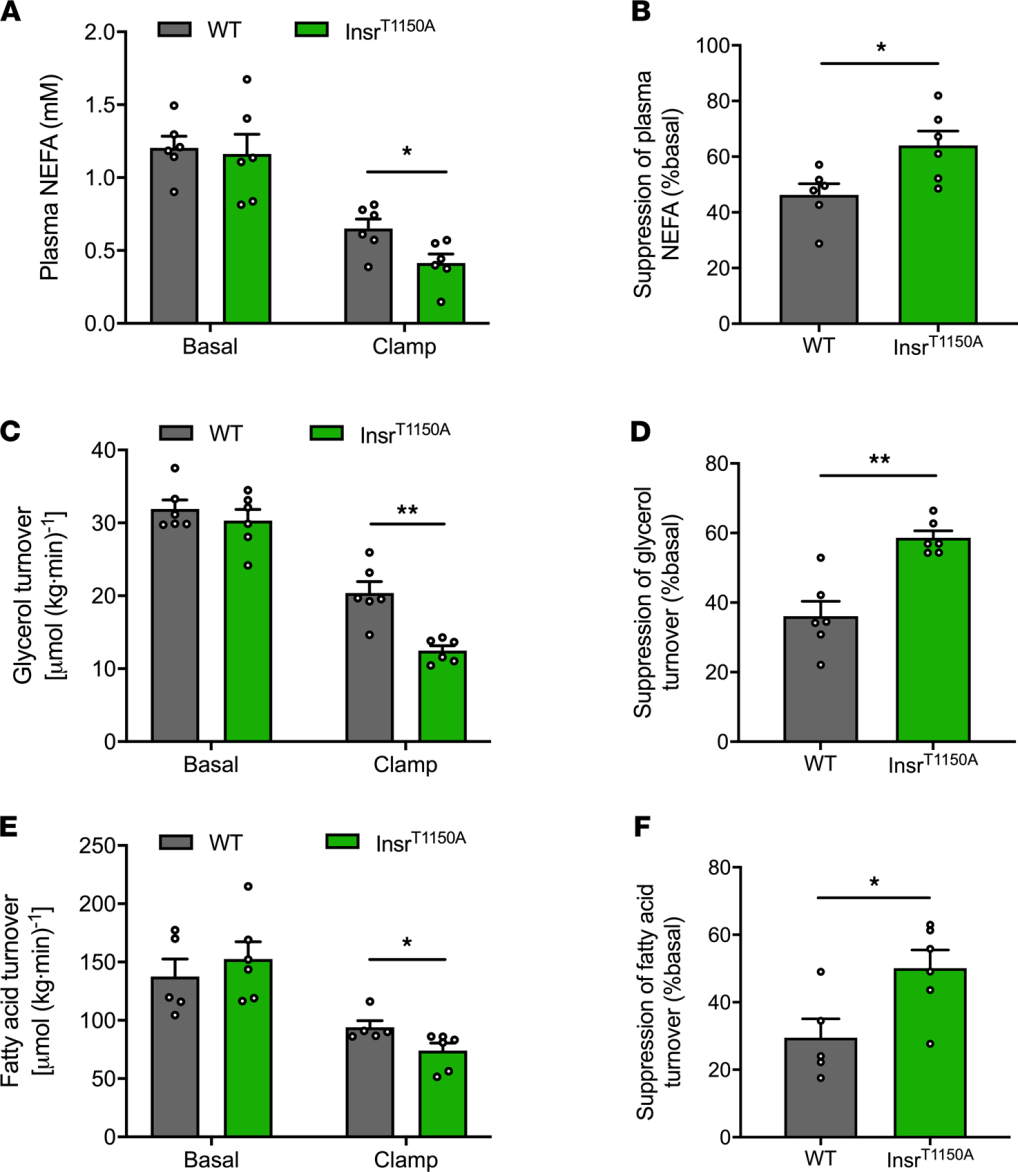
Insr^T1150A^ mice retain insulin’s ability to suppress WAT lipolysis after 7-day HFD. (**A**) Plasma NEFA under basal (overnight fasting) and hyperinsulinemic-euglycemic clamp conditions. (**B**) Insulin’s suppression of plasma NEFA during the hyperinsulinemic-euglycemic clamp. (**C** and **D**) Whole-body glycerol turnover and its suppression by insulin during the hyperinsulinemic-euglycemic clamp. (**E** and **F**) Whole-body fatty acid turnover and its suppression by insulin during the hyperinsulinemic-euglycemic clamp. In all panels, data are the mean ± SEM of *n* = 5–6 per group, with comparisons by 2-tailed unpaired Student’s *t* test. *P < 0.05, **P < 0.01.

**Figure 4:**
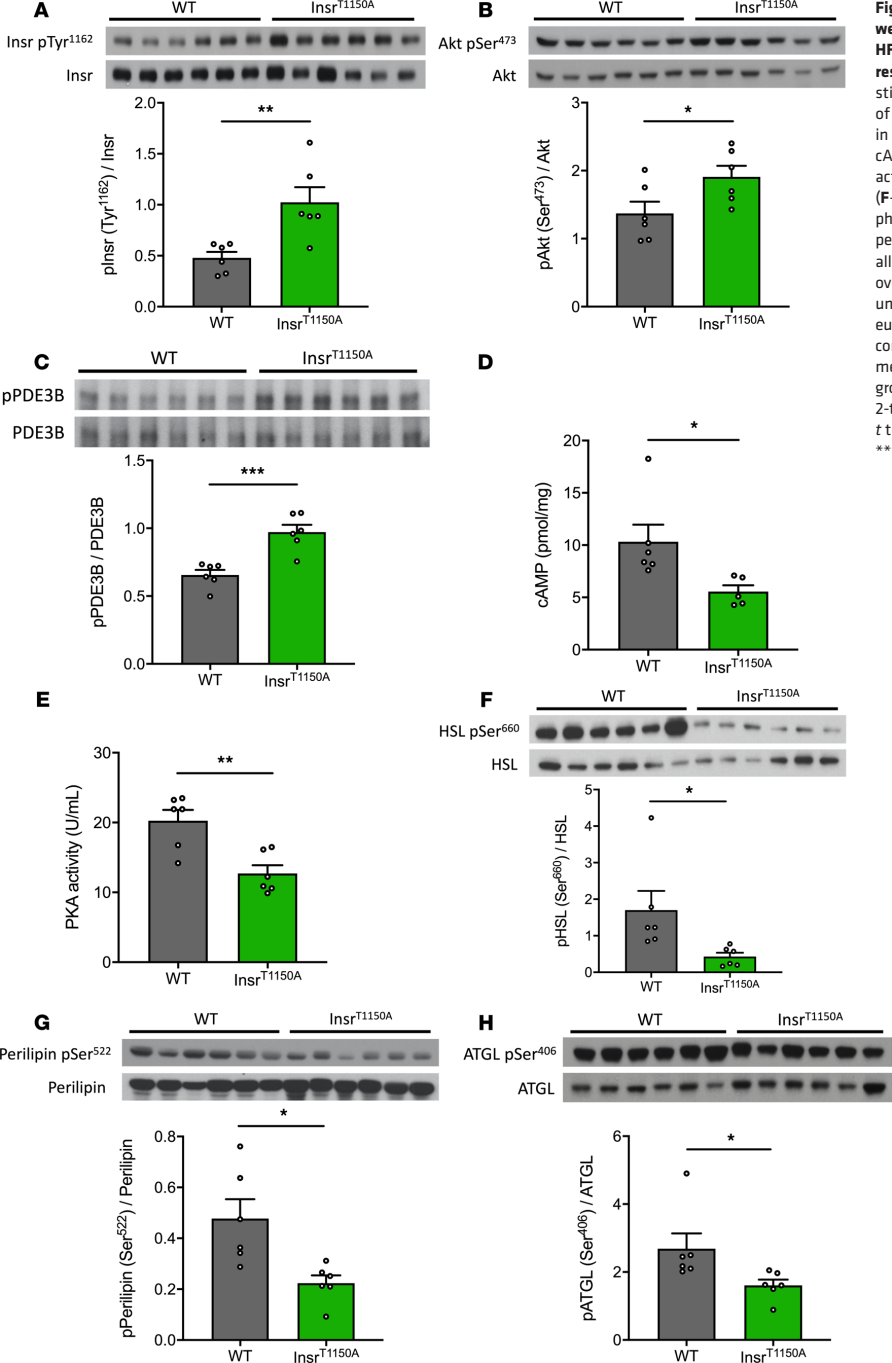
Insr^T1150A^ mice were protected from HFD-induced WAT insulin resistance. (**A**–**C**) Insulin-stimulated phosphorylation of Insr, Akt, and PDE3B in WAT. (**D** and **E**) WAT cAMP content and PKA activity during the clamp. (**F**–**H**) Insulin-stimulated phosphorylation of HSL, perilipin, and ATGL. In all panels, mice (after overnight fasting) were under hyperinsulinemic-euglycemic clamp condition; data are the mean ± SEM of *n* = 6 per group, with comparisons by 2-tailed unpaired Student’s *t* test. *P < 0.05, **P < 0.01, ***P < 0.001.

In order to address this possibility, we performed a much shorter 30-minute HEC study with a lower-dose insulin infusion rate (2.0 mU/[kg-min]) to evaluate insulin action in WAT in Insr^T1150A^ mice subjected to 7-day HFD. As observed previously, there were no significant differences in body composition, overnight fasting plasma glucose, insulin and NEFA concentrations, or whole-body rates of WAT lipolysis between the WT and Insr^T1150A^ mice (Figure 3, A, C, and E; Supplemental Figure 4, A–F; and Supplemental Table 2). Nevertheless, Insr^T1150A^ mice retained the ability of insulin to suppress WAT lipolysis, as reflected by lower plasma NEFA concentrations, whole-body glycerol turnover, and fatty acid turnover (Figure 3, A–F, and Supplemental Figure 4G) during the HEC. In addition, WAT insulin signaling was preserved in Insr^T1150A^ mice, reflected by higher insulin-stimulated Insr Tyr^1162^ phosphorylation and Akt Ser^473^ phosphorylation compared with HFD-fed WT mice (Figure 4, A and B). We also examined the impact of 7-day HFD feeding on the downstream proteins that regulate WAT lipolysis. In contrast to the WT mice subjected to 7-day HFD feeding, Insr^T1150A^ mice displayed increased PDE3B activity, which subsequently resulted in reduced cAMP levels and thereby decreased PKA activity (Figure 4, C–E). Consequently, phosphorylation of perilipin, HSL, and ATGL decreased in Insr^T1150A^ mice (Figure 4, F–H), consistent with the preservation of insulin-mediated suppression of WAT lipolysis. These data demonstrate that phosphorylation of Insr Thr^1160^ is required for the development of WAT insulin resistance after a 7-day HFD. Taken together with our prior studies (6–15), these findings suggest that lipid-induced liver, muscle, and WAT insulin resistance develop as a consequence of a common pathway involving increases in PM *sn*-1,2-DAG content leading to PKCε activation and the consequent impairment of IRK activation due to increased Insr Thr^1160^ phosphorylation.

## Discussion

Appropriate energy storage and release in healthy WAT is critical for survival under calorie-scarce conditions, as well as for proper nutrient distribution during feeding. Under conditions of overnutrition, this process is dysregulated, especially in individuals with inherited predisposition to restrained adipocyte capacity (23). Insulin regulates energy storage in WAT, in part by suppressing lipolysis. Dysregulated WAT lipolysis (along with a decreased capacity for adipocyte expansion) could promote ectopic lipid accumulation and, consequently, insulin resistance in liver and skeletal muscle (1, 4, 24–26). Increased WAT lipolysis will also increase hepatic gluconeogenesis by providing increased delivery of glycerol and NEFA to the liver, which both would, in turn, promote increased gluconeogenesis (1, 16). Therefore, insulin’s regulation of WAT lipolysis serves as a key component in the network of inter-organ communication and regulation of hepatic glucose production (1, 16). Defects in insulin’s suppression of WAT lipolysis may occur early in the transition from insulin sensitivity to resistance, with defects detectable as early as within 7–10 days of HFD feeding in rodents (27). This early defect precedes other well-characterized changes in the adipocytes (e.g., inflammation, hypoxia, necrosis), which in turn will also promote dysregulated WAT metabolism and increased lipolysis. The data presented here now provide a mechanistic underpinning for this phenomenon. Specifically, the PM *sn*-1,2-DAG/PKCε/Insr Thr^1160^ pathway that is responsible for lipid-induced hepatic insulin resistance, which occurs in MAFLD, may also account for lipid-induced WAT insulin resistance in the early stages of overnutrition.

Firstly, impaired WAT insulin signaling occurs rapidly during HFD feeding. We detected WAT insulin resistance after only 7 days of HFD feeding, with reduced insulin-mediated WAT glucose uptake and suppression of WAT lipolysis, which was accompanied by impaired activation of key insulin signaling steps initiating at the level of IRK tyrosine autophosphorylation. Importantly, this subtle development of WAT insulin resistance manifests earlier than other alterations such as inflammation and hypoxia in epididymal WAT, which may predispose WAT to more severe metabolic disturbances with prolonged HFD feeding.

WAT insulin resistance can be attributed to a proximal defect in insulin signaling at the level of the Insr, which ultimately impacts the regulation of key lipolytic enzymes. The defect in Insr activation leads to impaired Akt and PDE3B phosphorylation. As a consequence, the decrease in PDE3B activity permits higher concentrations of cAMP, which then promotes PKA activity. PKA directly phosphorylates and activates HSL (28, 29). PKA also phosphorylates perilipin, which promotes the release of CGI58 (a key coeffector of ATGL) and the recruitment of HSL to the LD (30). Some have proposed that insulin may regulate WAT lipolysis in an Akt-independent pathway. Choi et al. demonstrated a noncanonical Akt-independent, phosphoinositide-3 kinase–dependent pathway regulating WAT lipolysis by selectively altering PKA targets perilipin and HSL (31). This pathway would also be impacted by the proximal defect in Insr activation and, thus, is consistent with our proposed model of WAT insulin resistance.

Short-term HFD feeding increases the content of *sn*-1,2-DAGs in the PM of adipocytes. Though an adipocyte is mainly comprised of a massive LD, important signaling lipids are also present in other subcellular compartments. Here, we assessed the content of DAG stereoisomers in 5 subcellular compartments. As expected, the LD was the largest reservoir of DAGs, accounting for approximately 90% of total DAGs. However, in epididymal WAT, there was no difference in LD *sn*-1,2-DAG content following short-term HFD feeding. In contrast, the PM DAGs account for only approximately 1% of total DAGs in WAT, but HFD feeding caused an approximately 2-fold increase in PM *sn*-1,2-DAG content.

*sn*-1,2-DAGs are the primary DAG product of the reesterification pathway, while previous studies demonstrated that *sn*-2,3-DAG and *sn*-1,3-DAG are primarily generated through the lipolytic pathway (32). Therefore, a short-term HFD condition will likely promote more accumulation of *sn*-1,2-DAGs due to the increased flux of fatty acids into the reesterification pathway. As for the compartment specificity, it’s likely that, in short-term HFD (e.g., 7-day), the other membrane compartments — ER and Mito — have the ability to maintain a relatively steady pool of lipids, due to their relatively larger baseline lipid content or higher lipid handling capacity (oxidation or transport), while PM has a relatively small baseline lipid content and, therefore, is more prone to relatively large fold changes in *sn*-1,2-DAGs. In LDs, DAGs mostly originate from lipolysis, and since basal rates of lipolysis do not change under this condition, DAG content in LDs is not expected to change very much. However, in long-term (e.g., chronic HFD) conditions, DAG content will likely increase in the LD and potentially other subcellular compartments.

The 2-fold increase in PM *sn*-1,2-DAGs was associated with an approximately 2-fold increase in PKCε translocation, and PKCɛ has previously been implicated to play a role in WAT insulin action. PKCε antisense oligonucleotide treatment improved insulin-stimulated WAT glucose uptake in 3-day HFD–fed rats (5). Consistent with these results, Brandon et al. found that WAT-specific PKCɛ-KO mice displayed improved glucose tolerance on HFD, indicating that WAT PKCɛ activation may be an essential step in the development of WAT insulin resistance (21). However, there are some notable differences between the study of Brandon et al. and the present work. Brandon et al. reported that WAT-specific deletion of PKCε improved glucose tolerance in chronically HFD-fed mice, but they did not detect alterations in insulin’s regulation of WAT lipolysis or insulin-stimulated glucose uptake in WAT explant after 1 week of HFD. They also did not detect differences in plasma fatty acid concentrations during a glucose tolerance test. In contrast, our relatively low-dose HEC studies, combined with stable isotopic measurements of lipolytic rates, provided us with a more sensitive means to detect differences in insulin’s regulation of WAT lipolysis in vivo.

We have previously identified Insr Thr^1160^ as a specific target that could be phosphorylated by PKCε, leading to inhibition of IRK activity in the liver (6). Phosphorylation of the Thr^1160^ residue in the IRK activation loop is predicted to destabilize its active configuration, thereby inhibiting IRK activity. Therefore, Insr^T1150A^ mice are protected from HFD-induced hepatic insulin resistance (6). WAT is sensitive to the antilipolytic effects of insulin (33); thus, we were most likely unable to detect differences in WAT insulin action due to saturating dose of insulin administered in our prior HEC studies in the Insr^T1150A^ mice. In order to address this issue, we used a lower insulin infusion rate (2.0 mU/[kg-min] versus 2.5 mU/[kg-min]) for a shorter time (30 minutes versus 140 minutes) in the current study to better assess WAT insulin action in WT versus Insr^T1150A^ mice. Under this experimental condition, we demonstrated that Insr^T1150A^ mice were protected from HFD-induced WAT insulin resistance. WAT insulin signaling was preserved at the level of IRK activity down through Akt phosphorylation, thereby preserving insulin’s ability to suppress phosphorylation of perilipin, HSL, and ATGL through regulating PDE3B activity and cAMP content. In summary, we demonstrated that PKCε-mediated Insr Thr^1160^ phosphorylation regulates lipid-induced WAT insulin resistance by inhibiting IRK activity.

Taken together, these data demonstrate that PM *sn*-1,2-DAG/PKCε/Insr Thr^1160^ phosphorylation is a critical pathway in the pathogenesis of short-term HFD–induced insulin resistance in WAT. Alterations in this pathway may occur during the early stage of WAT lipid overinflux, resulting in impaired insulin’s regulation of WAT lipolysis and WAT glucose uptake. Though the defect in WAT is subtle, it may be a necessary predisposition to more severe WAT dysfunction, as well as for ectopic lipid deposition and insulin resistance in liver and skeletal muscle in prolonged states of overnutrition. Furthermore, given that PKCε has many additional targets in addition to Insr Thr^1160^, it is likely that PM *sn*-1,2-DAG–induced PKCε activation will impact many additional targets that affect WAT metabolism independently of changes in IRK activity (34). Thus, longer-term studies are needed to investigate if eliminating WAT insulin resistance could abrogate other metabolic disturbances and whether these results translate to humans under conditions of short-term overnutrition and obesity. Moreover, these data identify the PM *sn*-1,2-DAG/PKCε/Insr Thr^1160^ phosphorylation pathway as a potential therapeutic target to treat metabolic dysfunctions that are associated with WAT insulin resistance.

## Methods

### Animals.

Male Sprague Dawley rats weighing approximately 250 g were obtained from Charles River Laboratories and were maintained on a 12-hour light/12-hour dark cycle. After 1 week of acclimation, rats underwent surgery for placement of polyethylene catheters in the common carotid artery (PE50 tubing, Instech Solomon) and the jugular vein (PE90 tubing, Instech), and they were then fed either a RC diet (Harlan Teklad, 2018; 18% calories from fat, 58% from carbohydrate, 24% from protein) or a HFD (Dyets, 112245; 59% calories from fat, 26% from carbohydrate, 15% from protein), ad libitum for 7 days.

Mice were generated and housed in the Yale Animal Resources Center under a 12-hour light/12-hour dark cycle and received ad libitum access to food and water. Insr^T1150A^ mice were generated as previously reported (6). Male mice were studied at 14–18 weeks of age. Catheters were placed in the jugular vein 7–9 days before the HEC. Mice were fed either a RC diet (Harlan Teklad, TD2018; 18% fat, 58% carbohydrate, and 24% protein) or a HFD (Research Diets, D12492; 60% fat, 20% carbohydrate, and 20% protein).

### HECs.

After either 7 days of regular or high-fat feeding, rats were fasted overnight. The rats underwent a basal intraarterial prime infusion of [1,2,3,4,5,6,6-^2^H_7_]glucose (1.5 mg/[kg-min]), [^13^C_16_]palmitate (0.5 mg/[kg-min]), and [1,1,2,3,3-^2^H_5_]glycerol (1.5 mg/[kg-min]) for 5 minutes, followed by a continuous infusion at rates of 0.15 mg/(kg-min), 0.05 mg/(kg-min), and 0.15 mg/(kg-min), respectively, for 115 minutes. Blood samples were taken from the jugular vein catheter at 100, 110, and 120 minutes of the basal infusion for measurement of lipid turnover. A short (30-minute) HEC was then conducted starting with a primed/continuous infusion of human insulin (prime 25 mU/[kg-min] for 5 minutes, continuously at 2.5 mU/[kg-min]) and a variable infusion of 20% dextrose to maintain euglycemia (110 mg/dL, approximately). Blood samples were drawn from the venous catheter at 10, 20, and 30 minutes of the clamp, with 20- and 30-minute time points to measure clamp lipid turnover. Plasma insulin levels at 120 minutes of basal infusion and 30 minutes of the clamp were measured by radioimmunoassay in the Yale Diabetes Research Center. Gas chromatography–mass spectrometry (GC/MS) was used to measure plasma glycerol and palmitate atom percent excess (APE), as we have described (16), as well as to calculate rates of lipolysis by correcting for the percentage of individual fatty acid content among the content of total fatty acids. Glycerol and palmitate turnover was calculated as: turnover = (tracer APE/plasma APE – 1) × infusion rate. Another set of 140-minute clamp studies was performed as previously described (5). A 200 μCi bolus of 2-deoxy-[1-^14^C]-glucose (PerkinElmer) was injected at 120 minutes to monitor tissue-specific insulin-stimulated glucose uptake. For the assay of WAT glucose uptake, WAT samples were homogenized, and the supernatants were transferred to an ion-exchange column to separate ^14^C-2-deoxyglucose-6-phosphate from 2-deoxy-[1-^14^C]-glucose as previously described (35). At the end of the clamp, rats were euthanized with pentobarbital sodium (150 mg/kg), and tissues were immediately harvested and frozen with tongs precooled in liquid N_2_. Tissues and plasma were stored at –80°C for subsequent analysis.

For the mouse clamp, study cohorts consisted of homozygous male Insr^T1150A^ mice and littermate male WT controls, which were generated by our group (6). After 7 days of high-fat feeding, mice were fasted overnight. Awake mice under gentle tail restraint were first infused with [^13^C_16_]palmitate (0.3 mg/[kg-min]) and [^2^H_5_]glycerol (0.075 mg/[kg-min]) for 120 minutes to measure lipid turnover. A short (30-minute) HEC was then performed beginning with a primed/continuous infusion of human insulin (prime 4.8 mU/[kg-min] for 3 minutes, continuously at 2.0 mU/[kg-min]) and a variable infusion of 20% dextrose to maintain euglycemia (110 mg/dL, approximately). At the end of the clamp, mice were euthanized with pentobarbital sodium (150 mg/kg), and tissues were immediately harvested and snap-frozen in liquid N_2_. Tissues and plasma were stored at –80°C for subsequent analysis.

### Biochemical analysis.

Plasma glucose concentrations were measured using the YSI Glucose Analyzer. Plasma insulin was measured by radioimmunoassay. Plasma NEFA concentration was measured spectrophotometrically using a Wako NEFA kit (Wako Diagnostics). cAMP was measured by a cAMP ELISA kit (Enzo Life Science) in accordance with manufacturer protocol. PKA activity was measured by a PKA colorimetric activity kit (Invitrogen).

### Immunoblotting and IP.

WAT lysates were prepared in RIPA buffer with protease inhibitors (cOmplete MINI; Roche) and phosphatase inhibitors (PhosSTOP; Roche). Protein was measured by the BCA assay (Pierce), and equal amount of protein extraction was mixed with sample buffer containing 5% β-mercaptoethanol. After running the samples in 4%–12% Tris-glycine gels (Novex), proteins were electrotransferred to Immobilon-P PVDF membranes (MilliporeSigma) by semidry transfer. Membranes were blocked in 5% BSA for 1 hour at room temperature and then probed overnight at 4°C with primary antibodies. The antibodies were obtained from Cell Signaling Technology (Insr, catalog 3025; Insr pTyr^1162^, catalog 3918; Akt, catalog 2920; Akt pSer^473^, catalog 4060; perilipin, catalog 9349; HSL, catalog 4107; HSL pSer^660^, catalog 4126; ATGL, catalog 2138; GAPDH, catalog 5174), Abcam (ATGL pSer^406^, catalog ab135093; NaK-ATPase, catalog ab7671; Calnexin, catalog ab22595; VDAC, catalog ab14734), Thermo Fisher Scientific (PDE3B, catalog, 14-1973-82), FabGennix International Incorporated (pPDE3B, catalog PPD3B-140AP), VALA Sciences (perilipin pSer^522^, catalog 4856), and BD Biosciences (PKCε, catalog 610086). Membranes were then washed in TBS-T and incubated for 1 hour at room temperature with secondary antibodies (Cell Signaling Technologies). After washing in TBS-T 3 times, antibody binding was detected by enhanced chemiluminescence (Pierce). Films were developed within the linear dynamic range of signal intensity and then were scanned for digital analysis. Densitometry was performed using ImageJ (NIH) software.

IP was performed using 1–2 mg protein from lysates prepared as described above; the lysate was incubated with antibody PDE3B (Thermo Fisher Scientific) overnight for 16 hours and then mixed with protein A/G agarose beads (Santa Cruz Biotechnology Inc.) for 4 hours. Immune complexes were washed extensively in lysis buffer and eluted in Laemmli buffer for immunoblot analysis.

### PKCε translocation assay.

The PKCε membrane/cytosol ratio in freeze-clamped WAT was assessed by Western blot as previously reported (36) with slight modifications and assumed to represent PKCε activity in WAT. Briefly, 400–500 mg WAT from rats fasted for 6 hours was homogenized in ice-cold buffer A containing 20 mM Tris-HCl (pH 7.4), 1 mM EDTA, 0.25 mM EGTA, and 250 mM sucrose with protease inhibitors (cOmplete MINI, Roche). Lysate was centrifuged (60 minutes; 100,000*g*; 4°C) to separate the membrane and cytosol from LD. The supernatant was saved as the cytosolic fraction. The pellet was washed once in ice-cold buffer A to remove all the LD and cytosol and was then resuspended in buffer B, containing 250 mM Tris-HCl (pH 7.4), 1 mM EDTA, 0.25 mM EGTA, and 2% Triton X-100 with protease inhibitors by sonication; it was incubated at 4°C for 45 minutes to solubilize membrane proteins and centrifuged (60 minutes; 100,000*g*; 4°C). The supernatant was saved as the membrane fraction. Equal amounts of protein were subjected to measure the PKCε membrane/cytosol ratio by immunoblotting.

### DAG subcellular fractionation assay.

Subcellular fractionation was performed as described previously (37) with modifications. A total of 300–350 mg epididymal adipose tissue was homogenized using buffer A (250 mM sucrose, 10 mM Tris [pH 7.4], 0.5 mM EDTA) in a Dounce-type tissue grinder (Kontes no. 21). All subsequent centrifugation steps were performed at 4°C. The homogenate was centrifuged at 17,000*g* in an SS-34 rotor for 15 minutes to separate pellet A and supernatant A (including the top lipid layer). Pellet A was washed by being resuspended in buffer A and centrifuged at 17,000*g* in an SS-34 rotor for 20 minutes. Pellet A was then resuspended in buffer A and layered on top of 1.12M sucrose solution in a 2 mL ultracentrifuge tube and was centrifuged in a TLS-55 rotor at 105,000*g* for 20 minutes to separate interface B and pellet B. Interface B was collected and resuspended with buffer A and then centrifuged in a TLA-100.2 rotor at 60,000*g* for 9 minutes to obtain pellet C. Pellet C was washed by being resuspended in buffer A and centrifuged in a TLA-100.2 rotor at 60,000*g* for 9 minutes and was saved as plasma membrane fraction. Pellet B was washed by being resuspended in buffer A and centrifuged at 17,000*g* in an SS-34 rotor for 15 minutes and was collected as mitochondrial fraction. Supernatant A was centrifuged at 390,000*g* in Ti-70.1 rotor for 75 minutes to separate pellet D, supernatant D (collected as cytosol fraction), and the top lipid layer (collected as lipid droplet fraction). Pellet D was washed by being resuspended in buffer A and centrifuged at 390,000*g* in Ti-70.1 rotor for 60 minutes and was collected as ER fraction. DAGs were extracted from the 5 fractions and measured by liquid chromatography–MS/MS as described previously (27).

### Statistics.

Comparisons were performed using the 2-tailed Student’s *t* test, unpaired with significance defined as a *P* < 0.05. GraphPad Prism 8.0 was used for all statistical analysis. Data are presented as the mean ± SEM.

### Study approval.

All animal studies were approved by the Yale University IACUC and were performed in accordance with all regulatory standards.

## Author contributions

KL, RJP, VTS, and GIS designed the experimental protocols. DZ, KL, JS, and XL performed experiments. KL and DZ analyzed data. KL, VTS, and GIS wrote the manuscript, with input from all coauthors.

## Supplementary Material

Supplemental data

## Version 1 01/07/2021

In-Press Preview

## Version 2 02/22/2021

Electronic publication

## References

[1] Petersen MC, Shulman GI (2018). Mechanisms of insulin action and insulin resistance. Physiol Rev.

[2] Carobbio S (2017). Adipose tissue function and expandability as determinants of lipotoxicity and the metabolic syndrome. Adv Exp Med Biol.

[3] Bodis K, Roden M (2018). Energy metabolism of white adipose tissue and insulin resistance in humans. Eur J Clin Invest.

[4] Shulman GI (2000). Cellular mechanisms of insulin resistance. J Clin Invest.

[5] Samuel VT (2007). Inhibition of protein kinase Cepsilon prevents hepatic insulin resistance in nonalcoholic fatty liver disease. J Clin Invest.

[6] Petersen MC (2016). Insulin receptor Thr1160 phosphorylation mediates lipid-induced hepatic insulin resistance. J Clin Invest.

[7] Lyu K (2020). A membrane-bound diacylglycerol species induces PKCϵ-mediated hepatic insulin resistance. Cell Metab.

[8] Yu C (2002). Mechanism by which fatty acids inhibit insulin activation of insulin receptor substrate-1 (IRS-1)-associated phosphatidylinositol 3-kinase activity in muscle. J Biol Chem.

[9] Szendroedi J (2014). Role of diacylglycerol activation of PKCθ in lipid-induced muscle insulin resistance in humans. Proc Natl Acad Sci U S A.

[10] Song JD (2020). Dissociation of muscle insulin resistance from alterations in mitochondrial substrate preference. Cell Metab.

[11] Perry RJ (2018). Mechanisms by which a very-low-calorie diet reverses hyperglycemia in a rat model of type 2 diabetes. Cell Metab.

[12] Li X, et al. Mechanisms by which adiponectin reverses high fat diet-induced insulin resistance in mice [published online December 8, 2020]. Proc Natl Acad Sci U S A . 10.1073/pnas.1922169117 PMC7768680 33293421

[13] Perry RJ (2013). Reversal of hypertriglyceridemia, fatty liver disease, and insulin resistance by a liver-targeted mitochondrial uncoupler. Cell Metab.

[14] Perry RJ (2015). Controlled-release mitochondrial protonophore reverses diabetes and steatohepatitis in rats. Science.

[15] Abulizi A (2017). A controlled-release mitochondrial protonophore reverses hypertriglyceridemia, nonalcoholic steatohepatitis, and diabetes in lipodystrophic mice. FASEB J.

[16] Perry RJ (2015). Hepatic acetyl CoA links adipose tissue inflammation to hepatic insulin resistance and type 2 diabetes. Cell.

[17] Lee YS (2014). Increased adipocyte O2 consumption triggers HIF-1α, causing inflammation and insulin resistance in obesity. Cell.

[18] Caprio S (2017). Adolescent obesity and insulin resistance: roles of ectopic fat accumulation and adipose inflammation. Gastroenterology.

[19] Tam CS (2010). Short-term overfeeding may induce peripheral insulin resistance without altering subcutaneous adipose tissue macrophages in humans. Diabetes.

[20] Alligier M (2012). Subcutaneous adipose tissue remodeling during the initial phase of weight gain induced by overfeeding in humans. J Clin Endocrinol Metab.

[21] Brandon AE (2019). Protein kinase c epsilon deletion in adipose tissue, but not in liver, improves glucose tolerance. Cell Metab.

[22] Rando RR, Young N (1984). The stereospecific activation of protein kinase C. Biochem Biophys Res Commun.

[23] Lotta LA (2017). Integrative genomic analysis implicates limited peripheral adipose storage capacity in the pathogenesis of human insulin resistance. Nat Genet.

[24] Kahn BB (2019). Adipose tissue, inter-organ communication, and the path to type 2 diabetes: the 2016 banting medal for scientific achievement lecture. Diabetes.

[25] Saponaro C (2015). The subtle balance between lipolysis and lipogenesis: a critical point in metabolic homeostasis. Nutrients.

[26] Vatner DF (2015). Insulin-independent regulation of hepatic triglyceride synthesis by fatty acids. Proc Natl Acad Sci U S A.

[27] Cantley JL (2013). CGI-58 knockdown sequesters diacylglycerols in lipid droplets/ER-preventing diacylglycerol-mediated hepatic insulin resistance. Proc Natl Acad Sci U S A.

[28] DiPilato LM (2015). The role of PDE3B phosphorylation in the inhibition of lipolysis by insulin. Mol Cell Biol.

[29] Morigny P (2016). Adipocyte lipolysis and insulin resistance. Biochimie.

[30] Zechner R (2012). FAT SIGNALS--lipases and lipolysis in lipid metabolism and signaling. Cell Metab.

[31] Choi SM (2010). Insulin regulates adipocyte lipolysis via an Akt-independent signaling pathway. Mol Cell Biol.

[32] Eichmann TO (2012). Studies on the substrate and stereo/regioselectivity of adipose triglyceride lipase, hormone-sensitive lipase, and diacylglycerol-O-acyltransferases. J Biol Chem.

[33] Kraegen EW (1985). Dose-response curves for in vivo insulin sensitivity in individual tissues in rats. Am J Physiol.

[34] Gassaway BM (2018). PKCε contributes to lipid-induced insulin resistance through cross talk with p70S6K and through previously unknown regulators of insulin signaling. Proc Natl Acad Sci U S A.

[35] Youn JH, Buchanan TA (1993). Fasting does not impaird insulin-stimulated glucose uptake but alters intracellular glucose metabolism in conscious rats. Diabetes.

[36] Samuel VT (2004). Mechanism of hepatic insulin resistance in non-alcoholic fatty liver disease. J Biol Chem.

[37] Bogan JS (2001). Insulin-responsive compartments containing GLUT4 in 3T3-L1 and CHO cells: regulation by amino acid concentrations. Mol Cell Biol.

