# Supplementary material for: Short-term overnutrition induces white adipose tissue insulin resistance through sn-1,2-diacylglycerol/PKCε/insulin receptor Thr1160 phosphorylation
Source: JCI Insight. 2021 Feb 22;6(4):e139946. doi: 10.1172/jci.insight.139946 (PMC7934919; doi:10.1172/jci.insight.139946)
Supplement: Supplemental data [file jciinsight-6-139946-s189.pdf]

Figure S1

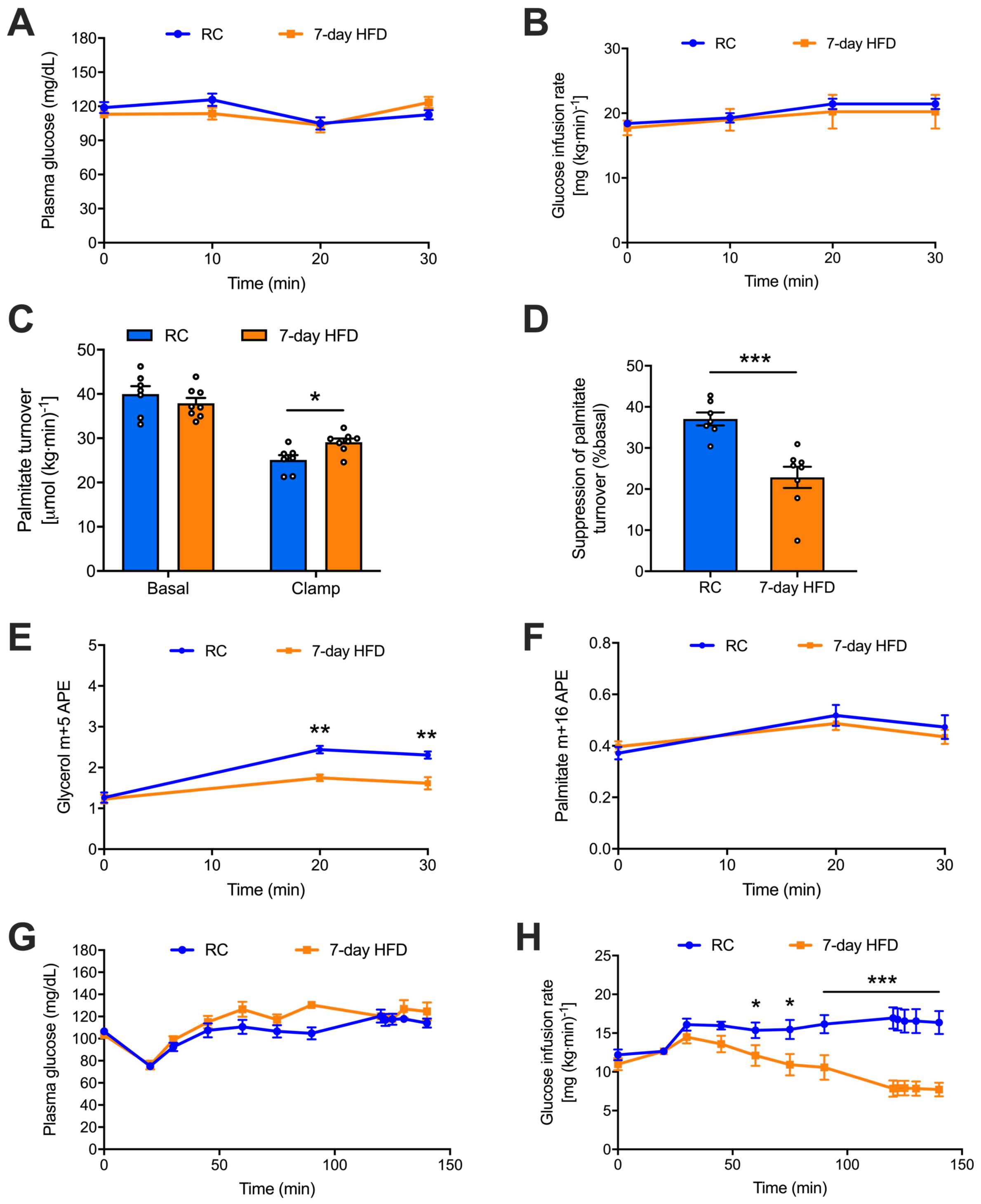

A
RC
7-day HFD
Plasma glucose (mg/dL)
Time (min)
B
Glucose infusion rate [mg (kg·min)-1]
Time (min)
C
Palmitate turnover [µmol (kg·min)-1]
Basal
Clamp
*
D
Suppression of palmitate turnover (%basal)
***
E
Glycerol m+5 APE
**
F
Palmitate m+16 APE
G
H
***

Figure S1. Hyperinsulinemic-euglycemic clamp studies in RC versus 7-day HFD rats

(A)-(B) Time course of plasma glucose and glucose infusion rates during a 30-min hyperinsulinemic-euglycemic clamp. (C)-(D) Whole-body palmitate turnover and its suppression by insulin during the clamp. (E)-(F) Time course of plasma glycerol m+5 APE and palmitate m+16 APE during the clamp. (G)-(H) Time course of plasma glucose and glucose infusion rates during a 140-min HEC. In all panels, data are the mean ± SEM of n = 6-10 per group, with comparisons by 2-tailed unpaired Student's t-test. *P<0.05, **P<0.01, ***P<0.001.

Figure S2

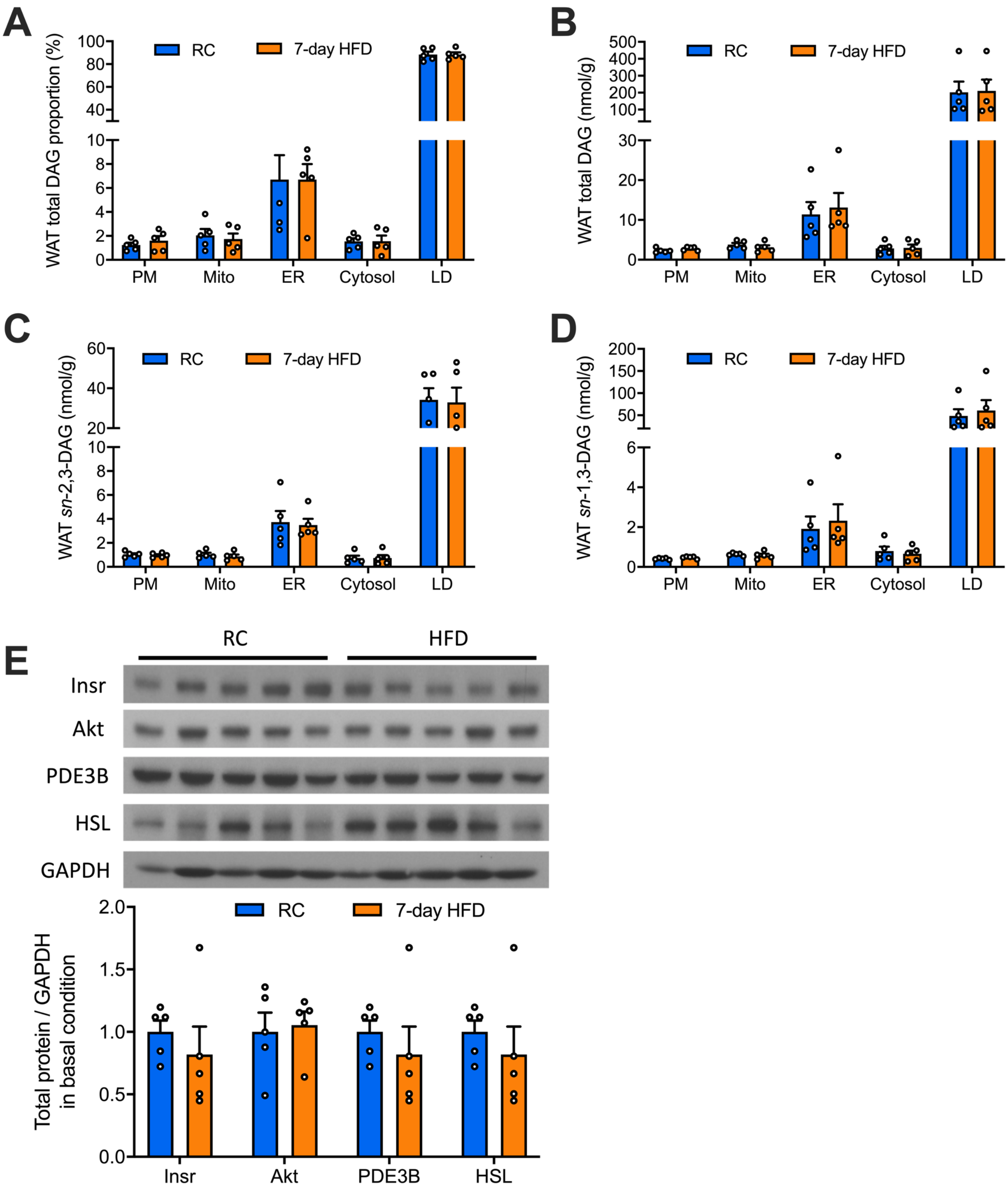

A
WAT total DAG proportion (%)
RC
7-day HFD
PM
Mito
ER
Cytosol
LD
B
WAT total DAG (nmol/g)
C
WAT sn-2,3-DAG (nmol/g)
D
WAT sn-1,3-DAG (nmol/g)
E
RC
HFD
Insr
Akt
PDE3B
HSL
GAPDH
Total protein / GAPDH in basal condition

Figure S2. DAG compartmental profile in WAT of RC versus 7-day HFD rats

(A) Total DAG proportion in five compartments. (B) WAT total DAG in five compartments. (C) WAT *sn*-2,3-DAGs in five compartments. (D) WAT *sn*-1,3-DAGs in five compartments. (E) WAT basal protein content. In all panels, rats were under basal condition (6-hr fasting), data are the mean ± SEM of n = 5 per group, with comparisons by 2-tailed unpaired Student's t-test.

Figure S3

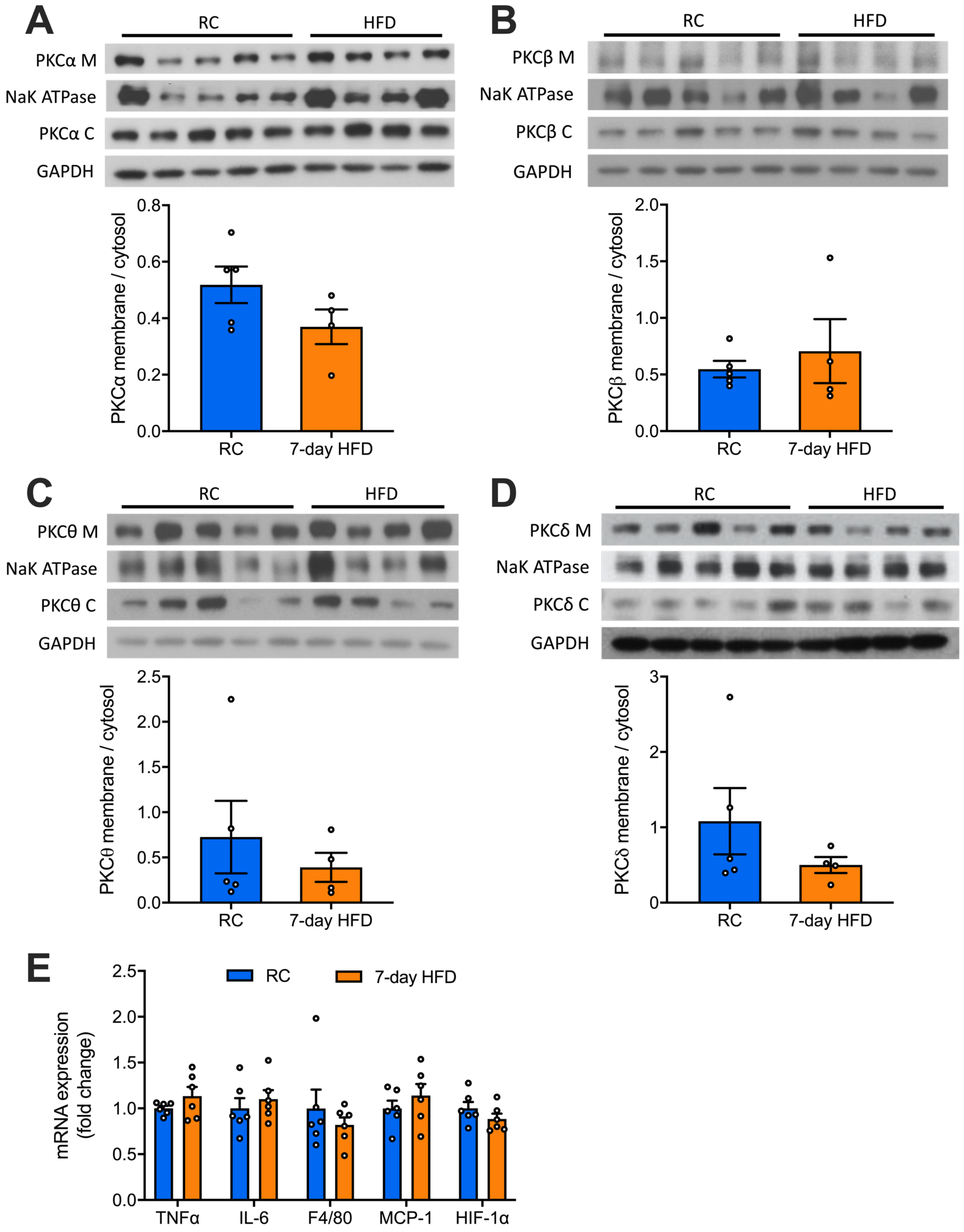
A
RC
HFD
PKCα M
NaK ATPase
PKCα C
GAPDH
PKCα membrane / cytosol
0.0
0.2
0.4
0.6
0.8
RC
7-day HFD
B
RC
HFD
PKCβ M
NaK ATPase
PKCβ C
GAPDH
PKCβ membrane / cytosol
0.0
0.5
1.0
1.5
2.0
RC
7-day HFD
C
RC
HFD
PKCθ M
NaK ATPase
PKCθ C
GAPDH
PKCθ membrane / cytosol
0.0
0.5
1.0
1.5
2.0
2.5
RC
7-day HFD
D
RC
HFD
PKCδ M
NaK ATPase
PKCδ C
GAPDH
PKCδ membrane / cytosol
0
1
2
3
RC
7-day HFD
E
RC
7-day HFD
mRNA expression
(fold change)
0.0
0.5
1.0
1.5
2.0
2.5
TNFα
IL-6
F4/80
MCP-1
HIF-1α

Figure S3. Translocation of PKCα, PKCβ, PKCθ, PKCδ and gene expression related to inflammation and hypoxia in WAT of RC versus 7-day HFD rats

(A) WAT PKCα membrane/cytosol ratio. (B) WAT PKCβ membrane/cytosol ratio. (C) WAT PKCθ membrane/cytosol ratio. (D) WAT PKCδ membrane/cytosol ratio. (E) Expression of genes associated with WAT inflammation and hypoxia. In all panels, rats were under basal condition (6-hr fasting), data are the mean ± SEM of *n* = 4-6 per group, with comparisons by 2-tailed unpaired Student's t-test.

Figure S4

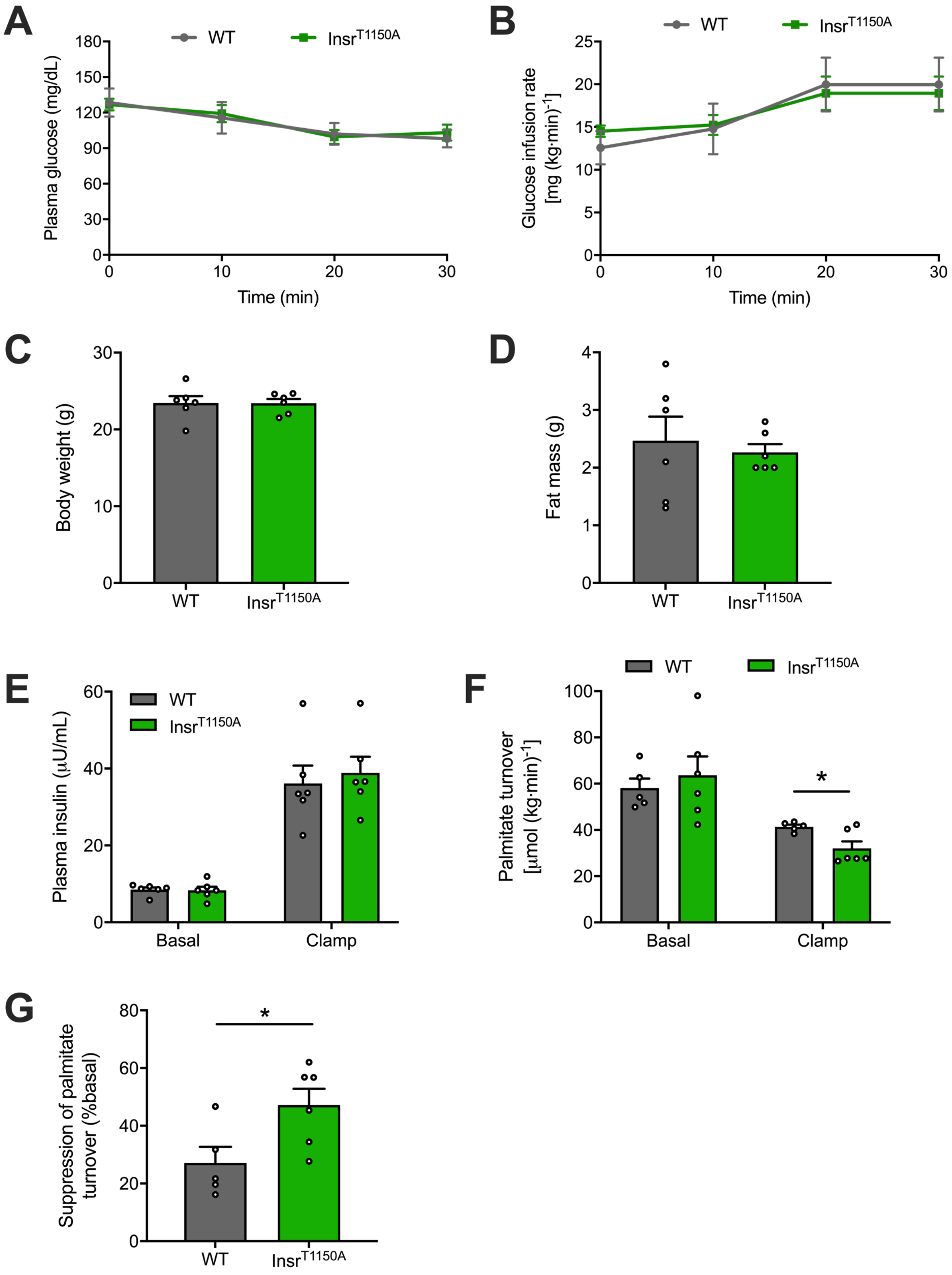

A
WT
Insr^{T1150A}
Plasma glucose (mg/dL)
180
150
120
90
60
30
0
0
10
20
30
Time (min)
B
WT
Insr^{T1150A}
Glucose infusion rate
[mg (kg·min)$^{-1}$]
25
20
15
10
5
0
0
10
20
30
Time (min)
C
Body weight (g)
30
20
10
0
WT
Insr^{T1150A}
D
Fat mass (g)
4
3
2
1
0
WT
Insr^{T1150A}
E
WT
Insr^{T1150A}
Plasma insulin (μU/mL)
60
40
20
0
Basal
Clamp
F
WT
Insr^{T1150A}
Palmitate turnover
[μmol (kg·min)$^{-1}$]
100
80
60
40
20
0
*
Basal
Clamp
G
Suppression of palmitate
turnover (%basal)
80
60
40
20
0
*
WT
Insr^{T1150A}

Figure S4. Hyperinsulinemic-euglycemic clamp studies in WT versus Insr^{T1150A} mice

(A)-(B) Time course of plasma glucose and glucose infusion rates. (C)-(D) Body weight and fat mass. (E) Plasma insulin concentrations under basal and clamp conditions. (F)-(G) Whole-body palmitate turnover and its suppression by insulin during the hyperinsulinemic-euglycemic clamp. In all panels, data are the mean ± SEM of n = 6 per group, with comparisons by 2-tailed unpaired Student's t-test. *P<0.05.

Table S1. Characteristics of rats in hyperinsulinemic-euglycemic clamp cohort

Parameters	RC	7-day HFD
Body weight (g)	309.0 ± 4.6	315.9 ± 4.5
Basal plasma glucose (mg/dL)	118.9 ± 4.80	113.0 ± 2.5
Basal plasma insulin (μU/mL)	7.3 ± 1.5	9.7 ± 1.3
Clamp plasma glucose (mg/dL)	112.6 ± 4.1	123.3 ± 5.0
Clamp plasma insulin (μU /mL)	75.7 ± 4.9	78.5 ±7.4

Data are presented as mean ± SEM of *n* = 8-10 per group, with comparisons by the 2-tailed unpaired Student's t-test.

Table S2. Characteristics of Insr^{T1150A} mice in hyperinsulinemic-euglycemic clamp cohort

Parameters	WT	Insr^{T1150A}
Body weight (g)	24.4 ± 0.9	23.6 ± 0.4
Fat mass (g)	2.7 ± 0.4	2.4 ± 0.2
Basal plasma glucose (mg/dL)	128.5 ± 11.9	126.8 ± 4.9
Basal plasma insulin (μU/mL)	8.5 ± 0.6	8.3 ± 0.9
Clamp plasma glucose (mg/dL)	98 ± 7.4	103 ± 6.8
Clamp plasma insulin (μU/mL)	36.1 ± 4.7	38.9 ± 4.2

Data are presented as mean ± SEM of *n* = 6 per group, with comparisons by the 2-tailed unpaired Student's t-test.

Table S3. Content of WAT *sn*-1,2-DAG individual species in RC- versus 7-day HFD-fed rats

sn-1,2-DAGs (nmol/g)	PM			Mito			ER		
**Species**	**RC**	**HFD**	***P***	**RC**	**HFD**	***P***	**RC**	**HFD**	***P***
**C16:0 C16:0**	0.077 ± 0.008	0.166 ± 0.018	0.004	0.220 ± 0.008	0.205 ± 0.062	0.789	0.814 ± 0.039	0.854 ± 0.234	0.855
**C16:0 C18:2**	0.155 ± 0.006	0.349 ± 0.031	0.001	0.451 ± 0.032	0.436 ± 0.095	0.874	1.846 ± 0.570	2.525 ± 0.985	0.550
**C18:1 C16:0**	0.065 ± 0.004	0.137 ± 0.011	0.001	0.188 ± 0.002	0.169 ± 0.045	0.654	0.689 ± 0.178	0.864 ± 0.381	0.667
**C18:0 C16:0**	0.020 ± 0.003	0.031 ± 0.002	0.022	0.033 ± 0.001	0.035 ± 0.007	0.739	0.102 ± 0.004	0.122 ± 0.030	0.487
**C16:0 C20:4**	0.003 ± 0.000	0.005 ± 0.001	0.038	0.008 ± 0.002	0.005 ± 0.002	0.310	0.017 ± 0.004	0.018 ± 0.003	0.810
**C18:2 C18:2**	0.028 ± 0.003	0.111 ± 0.021	0.007	0.138 ± 0.019	0.160 ± 0.018	0.447	0.620 ± 0.262	1.301 ± 0.482	0.229
**C18:1 C18:2**	0.063 ± 0.005	0.124 ± 0.018	0.019	0.196 ± 0.021	0.188 ± 0.028	0.818	0.721 ± 0.243	1.033 ± 0.371	0.489
**C18:1 C18:1**	0.083 ± 0.003	0.119 ± 0.016	0.080	0.183 ± 0.011	0.171 ± 0.042	0.778	0.631 ± 0.178	0.767 ± 0.278	0.680
**C18:2 C18:0**	0.024 ± 0.002	0.048 ± 0.004	0.003	0.053 ± 0.005	0.054 ± 0.007	0.855	0.177 ± 0.044	0.259 ± 0.078	0.363
**C18:1 C18:0**	0.016 ± 0.002	0.022 ± 0.001	0.035	0.028 ± 0.002	0.023 ± 0.005	0.380	0.105 ± 0.020	0.116 ± 0.038	0.800
**C18:0 C18:0**	0.131 ± 0.003	0.129 ± 0.005	0.711	0.111 ± 0.003	0.118 ± 0.003	0.175	0.130 ± 0.010	0.119 ± 0.007	0.451
**C18:0 C20:4**	0.013 ± 0.007	0.021 ± 0.009	0.562	0.013 ± 0.003	0.021 ± 0.005	0.189	0.036 ± 0.007	0.028 ± 0.004	0.386

sn-1,2-DAGs (nmol/g)	Cytosol			LD		
**Species**	**RC**	**HFD**	***P***	**RC**	**HFD**	***P***
**C16:0 C16:0**	0.134 ± 0.027	0.141 ± 0.036	0.883	5.294 ± 1.182	4.835 ± 1.247	0.799
**C16:0 C18:2**	0.284 ± 0.094	0.396 ± 0.202	0.606	21.89 ± 5.269	27.98 ± 6.847	0.496
**C18:1 C16:0**	0.138 ± 0.038	0.145 ± 0.049	0.915	8.339 ± 1.994	8.753 ± 2.270	0.894
**C18:0 C16:0**	0.022 ± 0.003	0.027 ± 0.004	0.307	0.729 ± 0.173	0.802 ± 0.240	0.807
**C16:0 C20:4**	0.005 ± 0.001	0.003 ± 0.001	0.135	0.108 ± 0.030	0.138 ± 0.031	0.514
**C18:2 C18:2**	0.065 ± 0.028	0.175 ± 0.094	0.256	8.132 ± 2.155	17.33 ± 4.614	0.093
**C18:1 C18:2**	0.075 ± 0.025	0.137 ± 0.071	0.390	7.727 ± 1.939	11.27 ± 2.905	0.328
**C18:1 C18:1**	0.088 ± 0.027	0.122 ± 0.036	0.473	7.821 ± 2.015	9.702 ± 2.860	0.597
**C18:2 C18:0**	0.036 ± 0.012	0.041 ± 0.017	0.785	1.594 ± 0.400	2.467 ± 0.680	0.283
**C18:1 C18:0**	0.015 ± 0.002	0.015 ± 0.005	0.995	1.051 ± 0.280	1.248 ± 0.392	0.687
**C18:0 C18:0**	0.118 ± 0.004	0.108 ± 0.004	0.130	0.312 ± 0.051	0.308 ± 0.054	0.960
**C18:0 C20:4**	0.011 ± 0.006	0.004 ± 0.001	0.385	0.058 ± 0.016	0.079 ± 0.019	0.414

Data are presented as mean ± SEM of *n* = 4-5 per group, with comparisons by the 2-tailed unpaired Student's t-test.
